# Predictors of Response to Corticosteroid Therapy in Idiopathic Granulomatous Mastitis: A Retrospective Cohort Study of 176 Patients

**DOI:** 10.3390/medicina62101915

**Published:** 2026-10-03

**Authors:** Cihan Atar, Kubilay Dalci, Burak Yavuz, Ali Yildirim, Tugba Toyran, Melek Ergin, Figen Binokay, Ahmet Gokhan Saritas, Atilgan Tolga Akcam, Gurhan Sakman

**Affiliations:** 1 Department of General Surgery, Osmaniye Training and Research Hospital, 80010 Osmaniye, Turkey; cihanatar@hotmail.com; 2 Department of General Surgery, Faculty of Medicine, Cukurova University, 01330 Adana, Turkey; kubilaydalci@hotmail.com (K.D.);; 3 Department of General Surgery, Koc University Hospital, 34010 Istanbul, Turkey; 4 Department of Pathology, Faculty of Medicine, Cukurova University, 01330 Adana, Turkey; 5 Department of Radiology, Faculty of Medicine, Cukurova University, 01330 Adana, Turkey

**Keywords:** idiopathic granulomatous mastitis, corticosteroid therapy, treatment response, predictive factors, breast disease

## Abstract

*Background and Objectives:* Idiopathic granulomatous mastitis (IGM) is a rare benign inflammatory breast disease with variable response to corticosteroid therapy. This study aimed to identify predictive factors for steroid responsiveness in IGM patients. *Materials and Methods:* A retrospective analysis of 176 patients with IGM treated at Cukurova University Faculty of Medicine, Department of General Surgery, between 2011 and 2024 was conducted. Patients were divided into steroid-responsive (Group 1, n = 100) and non-responsive (Group 2, n = 76) groups. Demographic characteristics, clinical features, and treatment history were compared using the chi-square test, Mann–Whitney U test, and multivariate logistic regression analysis. *Results:* Mean age was 39.1 ± 7.6 years. Factors associated with steroid non-responsiveness included extramammary symptoms (40.8% vs. 26.0%, *p* = 0.038), recurrent disease (21.1% vs. 8.0%, *p* = 0.012), a higher number of previous attacks (median 2 vs. 1, *p* < 0.001), previous abscess drainage (31.6% vs. 14.0%, *p* = 0.005), previous surgical treatment (30.3% vs. 7.0%, *p* < 0.001), and longer steroid treatment duration (median 6 vs. 4 months, *p* = 0.006). Multivariate analysis identified recurrent disease (odds ratio [OR] = 0.283, *p* = 0.015), previous abscess drainage (OR = 0.307, *p* = 0.007), previous surgery (OR = 0.170, *p* = 0.001), and steroid treatment duration (OR = 0.880, *p* = 0.008) as independent predictors of poor steroid response. *Conclusions:* Steroid non-responsiveness in IGM is associated with disease recurrence, previous abscess drainage, previous surgical intervention, and prolonged steroid therapy. Alternative treatment modalities should be considered in patients with these characteristics.

## 1. Introduction

Idiopathic granulomatous mastitis (IGM), first described by Kessler and Wolloch in 1972, is a rare, benign, chronic inflammatory disease of the breast [1]. Its etiology remains uncertain, although autoimmune processes, hormonal factors, and infectious agents have been proposed as potential contributing factors [2,3]. The efficacy of corticosteroid therapy in IGM has been widely interpreted as supporting an autoimmune-mediated pathophysiological process [3]. IGM predominantly affects women of reproductive age and poses both diagnostic and therapeutic challenges, particularly because of its clinical and radiological resemblance to inflammatory breast carcinoma [4]. For this reason, the definitive diagnosis of IGM still relies on needle biopsy and histopathological examination. Its distinguishing features include the absence of malignant cells on biopsy and the presence of non-caseating granulomatous inflammatory changes [4].

The optimal treatment strategy for IGM remains controversial. The uncertain etiology of the disease complicates management strategies [5]. A variety of therapeutic approaches have been used, including observation, antibiotics, corticosteroids, immunosuppressive agents, and surgical excision [6,7]. Corticosteroid therapy has emerged as the first-line treatment option, achieving clinical remission in 50–87% of patients [8,9]. However, a substantial proportion of patients either fail to respond to steroid therapy or experience disease recurrence, highlighting the need for alternative treatment strategies [10].

Despite the widespread use of corticosteroids in the management of IGM, factors predicting treatment response remain poorly defined. Identifying patients who are likely to respond to steroid therapy could help optimize treatment selection, minimize unnecessary adverse effects, and improve clinical outcomes. The present study aimed to identify clinical and demographic factors associated with corticosteroid response in a large cohort of IGM patients.

## 2. Materials and Methods

### 2.1. Study Design and Patient Selection

This retrospective cohort study was conducted at the Department of General Surgery, Cukurova University Faculty of Medicine. The medical records of all patients diagnosed with IGM between January 2011 and December 2024 were reviewed retrospectively. The study protocol was approved by the Research Ethics Committee of Cukurova University Faculty of Medicine (Meeting No. 157, Decision No. 27; 18 July 2025).

Inclusion criteria were: (1) histopathologically confirmed diagnosis of IGM, (2) treatment with oral corticosteroids, (3) a minimum follow-up period of 6 months, and (4) complete medical records. Exclusion criteria were: (1) specific granulomatous mastitis (tuberculosis, sarcoidosis, fungal infections), (2) concomitant malignancy, (3) incomplete treatment, and (4) loss to follow-up.

### 2.2. Treatment Protocol

All patients received oral prednisolone as initial therapy. The starting dose was 32 mg/day (n = 90) or 64 mg/day (n = 86), depending on disease severity and clinician preference. The dose was gradually tapered according to clinical response, typically by 5–10 mg every 2–4 weeks.

### 2.3. Definition of Treatment Response

Patients were classified into two groups according to their response to the course of oral corticosteroid therapy evaluated in this study (hereafter, the index course):

- Group 1 (Steroid-responsive): complete clinical and radiological resolution of the disease with corticosteroid therapy alone.
- Group 2 (Steroid non-responsive): failure to achieve complete remission, disease progression during treatment, or the need for additional intervention (surgery, immunosuppressants) because of an inadequate response to the index course.

### 2.4. Primary and Secondary Outcomes

The primary outcome was the response to the index corticosteroid course, classified as steroid-responsive (Group 1) or steroid non-responsive (Group 2) according to the definitions given above. The secondary outcomes were (i) the comparison of demographic characteristics, reproductive history, clinical presentation, and treatment history between the two groups; (ii) the comparison of the initial prednisolone dose (32 vs. 64 mg/day) and the duration of steroid therapy between the groups; and (iii) the identification of independent predictors of steroid non-responsiveness using multivariate logistic regression analysis.

### 2.5. Data Collection

The following variables were extracted from the medical records:

- Demographic data: age, body mass index (BMI).
- Reproductive history: menopausal status, parity, duration of breastfeeding, history of oral contraceptive use.
- Clinical presentation: presenting symptoms, extramammary findings, laterality, localization.
- Disease characteristics: recurrent disease, number of previous attacks, time from symptom onset to treatment.
- Treatment history: previous antibiotics, steroids, surgical interventions, abscess drainage.
- Treatment details: steroid dose, treatment duration, adverse effects.

All treatment-history variables, including previous abscess drainage and surgical intervention, referred exclusively to treatments and procedures performed before the initiation of the index corticosteroid course (for example, during earlier disease episodes or before referral to our department). Surgery or immunosuppressive therapy required during or after the index course because of an inadequate response was considered part of the outcome definition (Group 2) and was not recorded as previous treatment.

### 2.6. Statistical Analysis

Statistical analysis was performed using SPSS version 25.0 (IBM Corp., Armonk, NY, USA). Continuous variables were expressed as mean ± standard deviation or median (interquartile range [IQR]), depending on the normality of distribution. Categorical variables were presented as frequencies and percentages.

Between-group comparisons were performed using Student’s *t*-test or the Mann–Whitney U test for continuous variables, and the chi-square test or Fisher’s exact test for categorical variables. Variables with *p* < 0.05 in univariate analysis were included in a multivariate logistic regression model to identify independent predictors of steroid non-responsiveness. Odds ratios (OR) with 95% confidence intervals (CI) were calculated. Steroid responsiveness (Group 1) was modeled as the outcome event; therefore, an OR below 1 indicates a lower likelihood of response to corticosteroid therapy. To examine the temporal relationship between previous treatments and the response to the index course, response rates were also compared between patients with and without previous surgery and between patients with and without previous abscess drainage. A *p*-value < 0.05 was considered statistically significant.

## 3. Results

### 3.1. Demographic and Clinical Characteristics

A total of 176 patients were included in the study, comprising 100 patients in Group 1 and 76 patients in Group 2 (Table 1). The mean age of the overall cohort was 39.1 ± 7.6 years, with no significant difference between Group 1 and Group 2 (39.15 ± 8.06 vs. 39.02 ± 6.97 years, *p* = 0.456). Similarly, the mean body mass index (BMI) was comparable between the groups (27.65 ± 2.71 vs. 27.87 ± 2.87 kg/m^2^, *p* = 0.302).

The majority of patients were premenopausal in both groups (86.0% in Group 1 vs. 78.9% in Group 2), with no statistically significant difference in menopausal status (*p* = 0.218). Smoking rates were higher in Group 1 compared to Group 2 (38.0% vs. 26.3%); however, this difference did not reach statistical significance (*p* = 0.102).

The prevalence of rheumatologic diseases was low and similar between the groups (3.0% vs. 1.3%, *p* = 0.458). Likewise, there were no significant differences in family history of IGM (10.0% vs. 7.9%, *p* = 0.630) or breast malignancy (15.0% vs. 15.8%, *p* = 0.886).

### 3.2. Reproductive History

Reproductive history parameters were comparable between the two groups (Table 2). There was no significant difference in the history of oral contraceptive use (17.0% in Group 1 vs. 13.2% in Group 2, *p* = 0.484). The median number of pregnancies was similar between the groups [2.0 (2.0–3.75) vs. 2.5 (2.0–3.0), *p* = 0.794], as were the median number of abortions [0.00 (0.00–1.00) in both groups, *p* = 0.705] and live births [2.00 (1.00–3.00) vs. 2.00 (2.00–3.00), *p* = 0.747]. A history of breastfeeding was highly prevalent in both groups (93.0% vs. 94.7%, *p* = 0.637), with a similar median duration of breastfeeding [24.00 (12.00–48.00) vs. 24.00 (15.25–48.00) months, *p* = 0.832]. Additionally, the rate of puerperal mastitis history did not differ significantly between Group 1 and Group 2 (27.0% vs. 34.2%, *p* = 0.302).

### 3.3. Clinical Presentation and Disease Characteristics

The distribution of presenting symptoms was similar between the two groups (*p* = 0.397; Table 3). The most common presenting complaint was a breast mass (48.0% in Group 1 vs. 52.6% in Group 2), followed by mastalgia (23.0% vs. 11.8%) and erythema (15.0% vs. 17.1%). An abscess at presentation was observed in 9.0% of patients in Group 1 and 14.5% in Group 2.

Extramammary manifestations were significantly more frequent in Group 2 compared to Group 1 (40.8% vs. 26.0%, *p* = 0.038). Similarly, recurrent disease was significantly more common in Group 2 (21.1% vs. 8.0%, *p* = 0.012). In line with this finding, the median number of previous attacks was significantly higher in Group 2 [2.00 (1.00–3.00) vs. 1.00 (1.00–2.00), *p* < 0.001]. There was no significant difference between the groups in terms of disease laterality (*p* = 0.864). However, a history of abscess drainage was significantly more frequent in Group 2 compared to Group 1 (31.6% vs. 14.0%, *p* = 0.005).

### 3.4. Previous Treatment History

Previous treatment modalities differed significantly between the two groups (Table 4). The use of antibiotics was more frequent in Group 2 compared to Group 1 (64.5% vs. 51.0%); however, this difference did not reach statistical significance (*p* = 0.074). In contrast, corticosteroid use was significantly higher in Group 2 (55.3% vs. 38.0%, *p* = 0.023). Similarly, a history of surgical intervention was markedly more common in Group 2 than in Group 1 (30.3% vs. 7.0%, *p* < 0.001). Conversely, patients without any prior treatment were significantly more frequent in Group 1 compared to Group 2 (36.0% vs. 19.7%, *p* = 0.018). When the cohort was stratified by treatment history before the index course, 7 of the 30 patients (23.3%) with previous surgical intervention responded to corticosteroid therapy, compared with 93 of the 146 patients (63.7%) without previous surgery (*p* < 0.001). Likewise, the response rate was 36.8% (14/38) among patients with previous abscess drainage and 62.3% (86/138) among those without (*p* = 0.005).

### 3.5. Current Treatment Characteristics and Outcomes

The initial corticosteroid doses were comparable between the two groups, with no significant difference observed in the distribution of patients receiving 32 mg/day or 64 mg/day (*p* = 0.570; Table 5). However, the duration of steroid therapy was significantly longer in Group 2 compared to Group 1. The median treatment duration was 6.00 (3.00–10.00) months in Group 2, whereas it was 4.00 (2.50–6.00) months in Group 1 (*p* = 0.006).

### 3.6. Multivariate Analysis of Predictors of Steroid Non-Responsiveness

Multivariate logistic regression analysis was performed to identify independent predictors of steroid non-responsiveness (Table 6). Recurrent disease emerged as a significant independent predictor, associated with a markedly lower likelihood of steroid responsiveness (OR = 0.283, 95% CI: 0.102–0.785, *p* = 0.015). Similarly, a history of abscess drainage was independently associated with a reduced steroid response (OR = 0.307, 95% CI: 0.130–0.724, *p* = 0.007). Previous surgical intervention was also independently associated with a significantly decreased likelihood of treatment response (OR = 0.170, 95% CI: 0.058–0.499, *p* = 0.001). In addition, longer steroid treatment duration was significantly associated with lower responsiveness (OR = 0.880, 95% CI: 0.802–0.967, *p* = 0.008). Extramammary symptoms, number of previous attacks, prior corticosteroid use, and absence of previous treatment were not found to be significant predictors of steroid response (*p* > 0.05 for all).

## 4. Discussion

IGM is a disease with an unpredictable clinical course and heterogeneous response to treatment, and its optimal management remains controversial. The present study represents one of the largest single-center analyses examining the predictors of corticosteroid response in IGM. Whereas previous studies have mainly focused on predictors of recurrence [4], data on factors predicting the initial response to corticosteroids remain limited. Our findings show that approximately 57% of patients achieved complete remission with corticosteroid therapy alone, which is consistent with the 50–87% rates reported in previous studies [8,9]. However, a considerable proportion of patients (43%) did not respond adequately to treatment, highlighting the need for reliable markers to guide initial treatment selection.

Although corticosteroids are widely accepted as first-line therapy for IGM, response rates are highly variable and recurrence rates may be substantial [6,11,12]. This variability has been attributed to factors such as disease severity, extent of inflammation, and underlying immunological mechanisms [13]. Demographic factors and reproductive history characteristics did not differ between groups in terms of steroid treatment response. The absence of an association between these features and treatment response suggests that clinical characteristics of the disease may be more important than demographic and reproductive factors in predicting outcomes. Some previous studies have reported associations between IGM and recent pregnancy, lactation, oral contraceptive use, and smoking [14]. More recently, oral contraceptive use and smoking were identified as independent predictors of the need for step-up therapy in a Singaporean cohort in which half of the patients required the addition of methotrexate after initial corticosteroid treatment [15], and oral contraceptive use was also associated with relapse in a small retrospective series [16]. In our study, these factors did not influence response to steroid therapy. This may be interpreted as indicating that although such features may contribute to the emergence of the disease, they do not appear to affect its clinical severity. Differences in patient populations, treatment regimens, and outcome definitions may also account for the discrepancies between studies.

In its early stages, IGM typically presents as a rapidly growing breast mass accompanied by breast swelling, erythema, and pain. As the disease progresses, most patients enter an abscess phase, during which abscess rupture may occur [17]. Wang et al. reported that initiating corticosteroid therapy in the early phase of the disease, particularly during the mass stage, was more effective than initiating it in later stages [17]. In our study, no significant association was found between presenting breast symptoms and treatment response. Although steroid non-responsiveness was more frequent among patients with extramammary symptoms in comparative analysis, this factor was not retained as a significant predictor in multivariate logistic regression analysis. Erythema nodosum accompanying IGM has been associated with a more aggressive disease course, including higher rates of fistula formation and recurrence [18]. In contrast, a recent cohort study found that patients with granulomatous mastitis, erythema nodosum, and arthritis syndrome responded rapidly to glucocorticoids but more often required treatment for longer than 6 months, and had a low recurrence rate after an adequate course [19]. Extramammary involvement may therefore reflect a more intense systemic inflammatory response rather than true steroid resistance, which could explain why this variable lost its significance after adjustment in our model.

Multivariate analysis identified four independent predictors of steroid non-responsiveness: recurrent disease, previous abscess drainage, previous surgical intervention, and prolonged steroid treatment duration. These findings have important clinical implications for patient counseling and treatment planning.

Recurrent disease was independently associated with a lower likelihood of steroid response (OR = 0.283). This suggests that patients with a history of IGM recurrence may harbor a more aggressive disease phenotype or have underlying factors that render them less responsive to anti-inflammatory therapy. Previous studies have reported recurrence rates ranging from 5% to 67% in IGM, with some suggesting that inadequate initial treatment may contribute to recurrence [20]. Consistent with our findings, disease flares independently predicted the need for step-up therapy in the Singaporean cohort [15], and the Turkish consensus report recommends that recurrent cases be reclassified to guide subsequent treatment planning [21]. Our findings support the consideration of alternative or combination therapies in patients presenting with recurrent disease.

Another notable finding of our study was the association between a history of abscess drainage and non-response to steroid therapy (OR = 0.307). Abscess formation likely represents a more severe inflammatory process or secondary bacterial infection, both of which may complicate the disease and reduce the efficacy of corticosteroids. This observation is consistent with previous studies suggesting that complicated IGM cases require multimodal treatment [22]. In line with our results, the presence of an abscess and of recurrent abscesses was associated with disease recurrence in a cohort of 120 patients [23], and the need for percutaneous aspiration independently predicted escalation to methotrexate in the Singaporean cohort [15].

A history of previous surgical intervention was also independently associated with a reduced likelihood of steroid response (OR = 0.170). Although this was the lowest odds ratio in the model, its wide confidence interval overlapped with those of recurrent disease and previous abscess drainage; therefore, these predictors should not be ranked by strength. Importantly, this variable included only surgical procedures performed before the index corticosteroid course, whereas surgery required after steroid failure formed part of the outcome definition. Analyzed in this way, only 23.3% of the patients with previous surgery responded to the index course, compared with 63.7% of those without previous surgery. Nevertheless, this association may still partly reflect confounding by disease severity, as patients who had required surgery during earlier episodes likely had more extensive or refractory disease. Alternatively, surgical intervention may alter local tissue integrity and inflammatory patterns, or post-surgical fibrosis may impair subsequent response to steroid therapy. Consistent with an unfavorable disease course after surgery, Lermi et al. reported a recurrence rate of 66.1% among patients who had undergone surgical intervention as part of their initial treatment [23]. In such patients, the early use of immunosuppressive agents such as methotrexate or reoperation may be considered, in line with the international and Turkish consensus recommendations for steroid-resistant disease [7,21,24].

Interestingly, longer steroid treatment duration was associated with worse outcomes in our study. This most likely reflects reverse causality: non-responsive patients were treated for longer periods before treatment failure was acknowledged. This finding underscores the importance of early response assessment and timely transition to alternative therapies in non-responsive patients. The optimal duration of steroid therapy is not clearly established in the literature. The general tendency is to continue therapy for 3–6 months [11,17]. In our study, the treatment duration of 4–6 months is consistent with current recommendations for IGM management. Furthermore, clinical resolution does not always coincide with radiological resolution: in a cohort of 89 patients who achieved clinical recovery with steroid therapy, recurrence occurred in 50% of those with a partial radiological response, compared with 5.9% of those with a complete radiological response [25]. In line with this, the Turkish consensus report recommends that treatment be discontinued only after both complete clinical and radiological response have been achieved [21], which is consistent with the definition of response used in our study.

Comparison of steroid doses showed no difference in efficacy between 32 mg/day and 64 mg/day of prednisolone. This finding supports initiating treatment with lower doses and reserving dose escalation for non-responsive patients. In a randomized trial, a high-dose tapering regimen of prednisolone achieved a higher remission rate (93.3% vs. 53.3%) and a lower recurrence rate than a fixed dose of 5 mg/day [26]. Because both starting doses used in our cohort were considerably higher than that low-dose regimen, our results suggest that increasing the initial dose beyond 32 mg/day may not provide additional benefit.

Taken together, these findings clearly indicate that corticosteroid response in IGM is multifactorial. Disease chronicity, previous treatments, and complications such as abscess formation play important roles in determining treatment outcomes. This highlights the need for patient-tailored, individualized treatment strategies rather than a uniform approach applied to all patients [21,24]. Closer follow-up, early response assessment, and planning of combination therapies when necessary are particularly important in patients who carry risk factors for steroid non-responsiveness.

### 4.1. Study Limitations

This study has several limitations. The retrospective design may introduce selection and information bias. Treatment protocols varied over the study period, and although the definition of treatment response was clinically meaningful, it may not fully capture patients with partial response. The limited availability of long-term follow-up data precluded evaluation of late recurrences. Additionally, standardized imaging protocols and inflammatory markers that could provide further prognostic information were not available. Although 176 patients represent a relatively large cohort for a rare disease, the sample size is still small for multivariate modeling. The number of non-responders relative to the number of variables entered into the model was limited, as reflected by the wide confidence intervals of some estimates, and the model was not validated in an independent cohort. Treatment-history variables may also act as markers of disease severity (confounding by indication); therefore, the observed associations should not be interpreted as causal. Finally, as this was a single-center study conducted at a tertiary referral center, our results may not be generalizable to other populations, healthcare settings, or treatment protocols, and they should be interpreted with caution until confirmed by multicenter prospective studies.

### 4.2. Clinical Implications

Based on our findings, we propose a risk-stratified approach to the management of IGM. Patients without risk factors (first presentation, no previous abscess drainage, no previous surgery) are suitable candidates for corticosteroid monotherapy. Patients with one or more risk factors should be informed about the increased likelihood of treatment failure and may benefit from closer monitoring, earlier response assessment, or initial combination therapy.

Future prospective studies should validate these predictive factors and investigate whether risk-adapted treatment strategies improve outcomes. Further investigation of biomarkers, standardized imaging criteria, and optimal treatment duration will enhance the personalized management of IGM.

## 5. Conclusions

Non-responsiveness to corticosteroid therapy in IGM is associated with recurrent disease, previous abscess drainage, previous surgical intervention, and prolonged treatment duration. These factors may help identify patients who are unlikely to respond to steroid monotherapy, thereby facilitating the earlier implementation of alternative treatment strategies. A risk-stratified approach may optimize treatment selection and improve clinical outcomes in this challenging clinical setting.

## Figures and Tables

**Table 1:** Demographic and Clinical Characteristics.

Characteristic	Group 1 (n = 100)	Group 2 (n = 76)	Total (n = 176)	*p*-Value
Age (years)	39.15 ± 8.06	39.02 ± 6.97	39.1 ± 7.6	0.456
BMI (kg/m^2^)	27.65 ± 2.71	27.87 ± 2.87	27.74 ± 2.77	0.302
Menopausal status				0.218
Premenopausal	86 (86.0%)	60 (78.9%)	146 (83.0%)	
Postmenopausal	14 (14.0%)	16 (21.1%)	30 (17.0%)	
Smoking	38 (38.0%)	20 (26.3%)	58 (33.0%)	0.102
Rheumatologic disease	3 (3.0%)	1 (1.3%)	4 (2.3%)	0.458
Family history of IGM	10 (10.0%)	6 (7.9%)	16 (9.1%)	0.630
Family history of breast malignancy	15 (15.0%)	12 (15.8%)	27 (15.3%)	0.886

BMI, body mass index; IGM, idiopathic granulomatous mastitis.

**Table 2:** Reproductive History.

Variable	Group 1 (n = 100)	Group 2 (n = 76)	Total (n = 176)	*p*-Value
OCP use history	17 (17.0%)	10 (13.2%)	27 (15.3%)	0.484
Number of pregnancies *	2.0 (2.0–3.75)	2.5 (2.0–3.0)	2.0 (2.0–3.0)	0.794
Number of abortions *	0.00 (0.00–1.00)	0.00 (0.00–1.00)	0.00 (0.00–1.00)	0.705
Number of live births *	2.00 (1.00–3.00)	2.00 (2.00–3.00)	2.00 (1.25–3.00)	0.747
Breastfeeding history	93 (93.0%)	72 (94.7%)	165 (93.8%)	0.637
Breastfeeding duration (months) *	24.00 (12.00–48.00)	24.00 (15.25–48.00)	24.00 (12.00–48.00)	0.832
Puerperal mastitis history	27 (27.0%)	26 (34.2%)	53 (30.1%)	0.302

* Data presented as median (IQR). IQR, interquartile range; OCP, oral contraceptive pill.

**Table 3:** Clinical Presentation and Disease Characteristics.

Variable	Group 1 (n = 100)	Group 2 (n = 76)	*p*-Value
Presenting symptom			0.397
Abscess	9 (9.0%)	11 (14.5%)	
Mastalgia	23 (23.0%)	9 (11.8%)	
Mass	48 (48.0%)	40 (52.6%)	
Erythema	15 (15.0%)	13 (17.1%)	
Nipple discharge	4 (4.0%)	3 (3.9%)	
Nipple retraction	1 (1.0%)	0 (0.0%)	
Extramammary symptoms	26 (26.0%)	31 (40.8%)	0.038
Recurrent disease	8 (8.0%)	16 (21.1%)	0.012
Number of previous attacks *	1.00 (1.00–2.00)	2.00 (1.00–3.00)	<0.001
Laterality			0.864
Right	36 (36.0%)	29 (38.2%)	
Left	60 (60.0%)	43 (56.6%)	
Bilateral	4 (4.0%)	4 (5.3%)	
Previous abscess drainage	14 (14.0%)	24 (31.6%)	0.005

* Data presented as median (IQR). IQR, interquartile range.

**Table 4:** Previous Treatment History.

Treatment	Group 1 (n = 100)	Group 2 (n = 76)	*p*-Value
Antibiotics	51 (51.0%)	49 (64.5%)	0.074
Corticosteroids	38 (38.0%)	42 (55.3%)	0.023
Surgery	7 (7.0%)	23 (30.3%)	<0.001
No previous treatment	36 (36.0%)	15 (19.7%)	0.018

**Table 5:** Current Treatment Characteristics and Outcomes.

Variable	Group 1 (n = 100)	Group 2 (n = 76)	*p*-Value
Steroid dose			0.570
32 mg/day	53 (53.0%)	37 (48.7%)	
64 mg/day	47 (47.0%)	39 (51.3%)	
Steroid duration (months) *	4.00 (2.50–6.00)	6.00 (3.00–10.00)	0.006

* Data presented as median (IQR). IQR, interquartile range.

**Table 6:** Multivariate Logistic Regression Analysis for Predictors of Steroid Non-responsiveness.

Variable	B	SE	OR	95% CI	*p*-Value
Extramammary symptoms	−0.197	0.421	0.821	0.360–1.872	0.639
Recurrent disease	−1.263	0.521	0.283	0.102–0.785	0.015
Number of previous attacks	−0.307	0.242	0.735	0.458–1.182	0.204
Previous abscess drainage	−1.180	0.437	0.307	0.130–0.724	0.007
Previous surgery	−1.772	0.550	0.170	0.058–0.499	0.001
Previous steroids	−0.303	0.447	0.739	0.308–1.774	0.498
No previous treatment	0.018	0.515	1.018	0.371–2.791	0.972
Steroid duration	−0.127	0.048	0.880	0.802–0.967	0.008
Constant	2.452	0.595	11.612	—	<0.001

Model statistics: −2 Log likelihood = 191.929; Cox & Snell R^2^ = 0.242; Nagelkerke R^2^ = 0.325; Hosmer–Lemeshow test *p* = 0.524. B, regression coefficient; SE, standard error; OR, odds ratio for steroid responsiveness (values below 1 indicate a lower likelihood of response); CI, confidence interval.

## Data Availability

The data presented in this study are available on request from the corresponding author due to privacy and ethical restrictions concerning patient confidentiality.

## Abbreviations

The following abbreviations are used in this manuscript:

BMI	Body mass index
CI	Confidence interval
IGM	Idiopathic granulomatous mastitis
IQR	Interquartile range
OCP	Oral contraceptive pill
OR	Odds ratio
SE	Standard error
SPSS	Statistical Package for the Social Sciences
